# The Stretcher Bearer

**Published:** 1915-12

**Authors:** 


**The Stretcher Bearer.** By GEORGES M. DUPUY. Pp. 138. Illustrated. London: Oxford Medical Publications. 1915.

Published as an addition to the Royal Army Medical Corps Training Book, this little pocket volume will prove of very great value to men who have had little experience of military or ambulance training, to enable them to form a clear idea of a command, as they can see the order actually carried out in the excellent series of illustrations. We consider it will certainly prove of great assistance to stretcher bearers especially.

